# Clinical Overview of MDM2/X-Targeted Therapies

**DOI:** 10.3389/fonc.2016.00007

**Published:** 2016-01-27

**Authors:** Andrew Burgess, Kee Ming Chia, Sue Haupt, David Thomas, Ygal Haupt, Elgene Lim

**Affiliations:** ^1^The Kinghorn Cancer Centre, Garvan Institute of Medical Research, Sydney, NSW, Australia; ^2^Faculty of Medicine, St. Vincent’s Clinical School, UNSW Australia, Sydney, NSW, Australia; ^3^The Sir Peter MacCallum Department of Oncology, the University of Melbourne, Melbourne, VIC, Australia

**Keywords:** p53, MDM2, MDMX, cancer therapy, nutlin

## Abstract

MDM2 and MDMX are the primary negative regulators of p53, which under normal conditions maintain low intracellular levels of p53 by targeting it to the proteasome for rapid degradation and inhibiting its transcriptional activity. Both MDM2 and MDMX function as powerful oncogenes and are commonly over-expressed in some cancers, including sarcoma (~20%) and breast cancer (~15%). In contrast to tumors that are p53 mutant, whereby the current therapeutic strategy restores the normal active conformation of p53, MDM2 and MDMX represent logical therapeutic targets in cancer for increasing wild-type (WT) p53 expression and activities. Recent preclinical studies suggest that there may also be situations that MDM2/X inhibitors could be used in p53 mutant tumors. Since the discovery of nutlin-3a, the first in a class of small molecule MDM2 inhibitors that binds to the hydrophobic cleft in the N-terminus of MDM2, preventing its association with p53, there is now an extensive list of related compounds. In addition, a new class of stapled peptides that can target both MDM2 and MDMX have also been developed. Importantly, preclinical modeling, which has demonstrated effective *in vitro* and *in vivo* killing of WT p53 cancer cells, has now been translated into early clinical trials allowing better assessment of their biological effects and toxicities in patients. In this overview, we will review the current MDM2- and MDMX-targeted therapies in development, focusing particularly on compounds that have entered into early phase clinical trials. We will highlight the challenges pertaining to predictive biomarkers for and toxicities associated with these compounds, as well as identify potential combinatorial strategies to enhance its anti-cancer efficacy.

## Introduction: Rationale for Targeting the p53 Pathway

**Figure 1:**
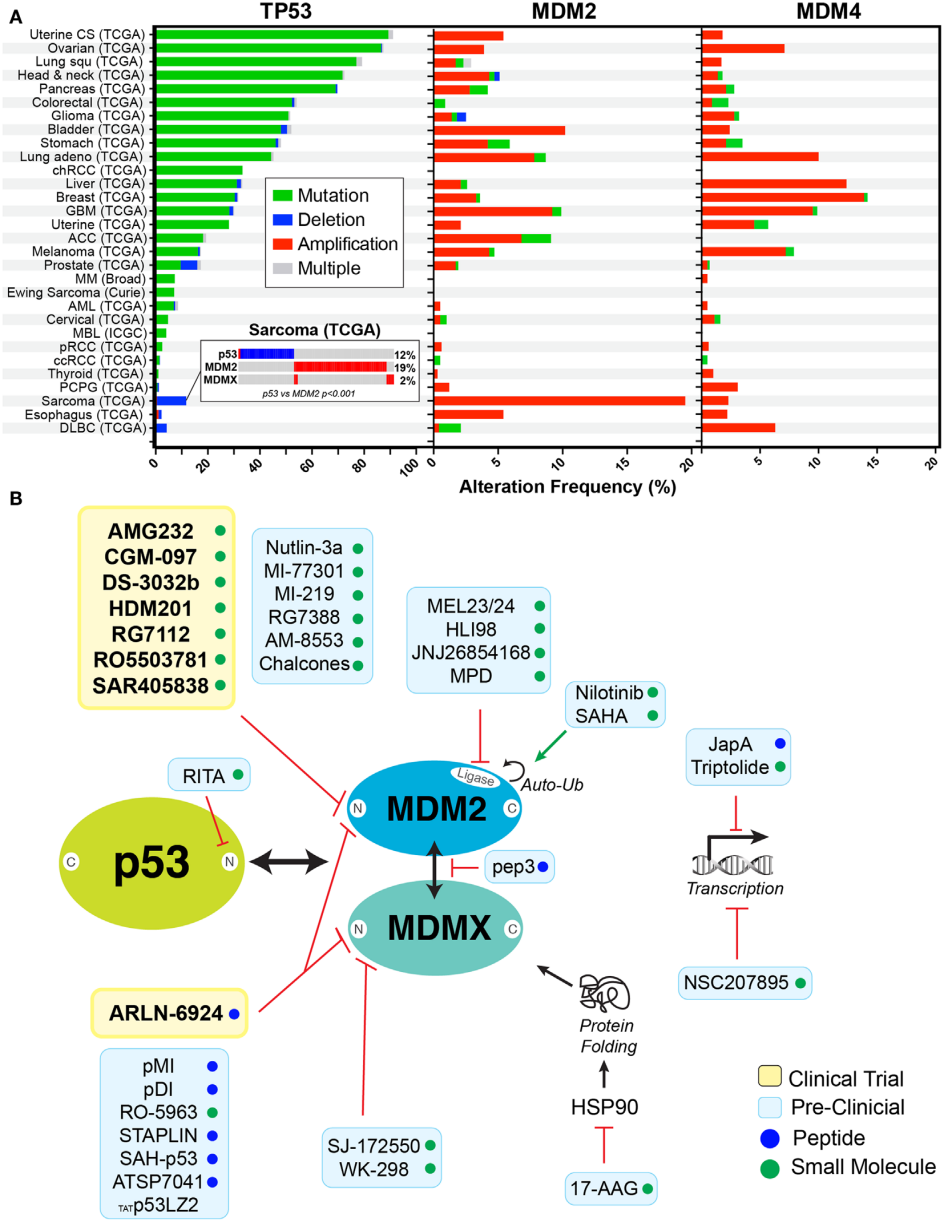
**Rationale for targeting p53 in cancers**. **(A)** Frequency of alterations are shown with mutation (green), deletion (blue), amplification (red), and combination of alterations (gray) in p53, MDM2, and MDMX in cancers derived from cBioPortal (5) (http://www.cbioportal.org). Insert shows the mutual exclusivity observed between MDM2 expression and p53 deletion in sarcomas. **(B)** Schematic representation of inhibitors in clinical trials (yellow box) or in preclinical studies (blue box) targeting the p53–MDM2/X axis. Compounds are either small molecules (green circle) or peptide (blue circle).

The tumor suppressor protein p53, nominated “the guardian of the genome,” is mutated in ~50% of all human cancers. However, the incidence of p53 mutations differs significantly between cancer types, ranging from near universal mutation (~96%) in serous ovarian cancer to rare occurrence (<10%) in thyroid cancer (Figure 1A). This disparity provides therapeutic opportunities for targeting cancers with p53 wild-type (WT), in a distinct manner from those with p53 mutant cancers. Several preclinical studies have demonstrated that reconfiguration of mutant, to its normal, active WT p53 conformation, restores apoptosis and promotes tumor regression (1–3). Therapeutic targeting of mutant p53, using small molecule drugs, is in the most advanced state for PRIMA-1, and its derivative PRIMA-1MET, an approach which restores the normal, active conformation of p53, which has been previously explored in depth by Wiman and coworkers (4). In the current review, we focus on therapies that target MDM2 and MDMX as a means of increase the stability of WT p53 and the consequences for patients with either WT p53 or mutant cancer cells.

### Regulation of p53 Stability by MDM2 and MDMX

The primary response to a variety of cellular insults and stresses is to concurrently activate and stabilize p53 within the cell. Activated p53 then drives a vast transcriptional program that arrests the cell cycle, promotes repair pathways, and in response to severe stress initiates apoptosis. Therefore, under normal conditions, it is critical that intracellular levels of p53 are kept low, which is achieved by the rapid degradation of p53 by the proteasome. This degradation occurs in both ubiquitin-dependent (6) and ubiquitin-independent mechanisms (7) and can be modulated by various signaling pathways including sumoylation, phosphorylation, acetylation, methylation, and glycosylation (8). Of these, ubiquitination is the most important (6, 9) and the E3 ligase MDM2 is the primary negative regulator of p53 (10, 11), although several other E3 and E4 ligases of p53 also exist (8, 9). Mechanistically, engagement of the p53 N-terminal transactivation domain by the N-terminal of MDM2, facilitates its C-terminal RING finger E3 ligase activity to transfer ubiquitin to multiple lysine residues of p53, located in central DNA-binding and C-terminal regulatory regions (8, 9). MDM2 ubiquitination of p53 (either mono- or poly-ubiquitination) negatively regulates its transcriptional activity. Mono-ubiquitin triggers nuclear export, while poly-ubiquitin targets nuclear p53 for degradation by the proteasome (12). Notably, the C-terminal of MDM2 is also able to bind with the C-terminal of the highly related protein MDMX (also known as HDMX and MDM4). Although MDMX does not possess E3 ligase activity, the MDM2–MDMX heterodimer ubiquitinates p53 with higher efficiency than MDM2 homodimers (13). MDMX, via its N-terminus, is able to bind p53 and efficiently inhibit its transcriptional activity (14). Furthermore, MDM2 is transcriptionally up regulated by p53 and this negative-feedback loop associated with cyclical modulation of levels of both proteins, ensures that p53 levels remain low under normal conditions (15).

### Targeting MDM2 and MDMX

Given the importance of both MDM2 and MDMX in regulating WT p53, it is unsurprising that they are commonly over-expressed in some cancers, including sarcoma (~20%) and breast (~15%) (Figure 1A). In this context, they function as powerful oncogenes and represent logical therapeutic targets for increasing WT p53 expression and activities. The concept of MDM2 targeting was supported by the discovery of p14^ARF^ (p19^ARF^ in mice), an alternate reading frame protein produced from the *CDKN2A* locus (16, 17). P14^ARF^ binds to MDM2, sequestering it in the nucleolus and preventing it from targeting p53 for degradation (18, 19). More precisely, the capacity to bind and sequester MDM2 to the nucleus was assigned to a 22 amino acid fragment from the N-terminus of p14^ARF^, revealing a potential method for targeting MDM2 with small peptide inhibitors (20). The first successful realization of this potential came in 2004, when nutlin-3a was discovered by Vassilev et al. (21). Nutlin-3a potently binds to the hydrophobic cleft in the N-terminus of MDM2, preventing its association with p53. Importantly, it is highly effective killing of WT p53 cancer cells, both *in vitro* and *in vivo* in preclinical models, provided validation for its use. However, its poor bioavailability, high toxicity (discussed in greater detail below), and its limited effects on MDMX overexpressing cells (22–24) has prevented its translation to the clinic. Recent interest has switched to compounds that have better bioavailability and can target both MDM2 and MDMX. These new compounds can be broadly segregated according to their mode of action. The vast majority of preclinical and clinical small molecule inhibitors work similarly to nutlin-3a, binding to the N-terminal pocket of MDM2, inhibiting association with p53 (Figure 1B). Despite the similarity in the N-terminal p53-binding domain of MDM2 and MDMX, most of these small molecule inhibitors bind with significantly less avidity to MDMX and are therefore primarily MDM2 specific (12). However, there are now several new peptide-based inhibitors that are capable of binding to the N-terminal of both MDM2 and MDMX (Table 1). In addition, several small molecule inhibitors, which bind specifically to the N-terminus of MDMX, have recently been developed and are currently undergoing preclinical testing (25, 26). In addition, there are now a growing number of new MDM2/X inhibitors that bind outside the N-terminus (Figure 1B). These include small molecules that inhibit the ubiquitin ligase activity of MDM2 (27); disruptors of MDM2–MDMX heterodimerization (28); transcriptional inhibitors of both *MDM2* (29, 30) and *MDMX* (31); MDM2 auto-ubiquitination activators (32, 33); inhibitors of HSP90 to disrupt MDMX protein folding; and molecules that directly engage p53 and prevent association with MDM2/X (34).

**Table 1:** **MDM2 and MDMX inhibitors in clinical development**.

MDM2 inhibitors in clinical development						

Class and specificity	Nature of compound	Compound	Status	p53	NCT identifier	Company
Small molecule MDM2 antagonists	Cis-imidazoline	RG7112	Phase I in advanced solid and hematological cancers, and liposarcoma (completed)	n/a	NCT00559533	
		RG7112 with cytarabine	Phase I in acute myelogenous leukemia (completed)	n/a	NCT01635296	
		RG7112 with doxorubicin	Phase I in soft tissue sarcoma (completed)	n/a	NCT01605526	Roche
		RO5503781	Phase I in advanced solid cancers (completed)	n/a	NCT01462175	
		RO5503781 with cytarabine	Phase I in acute myelogenous leukemia (active but not recruiting)	n/a	NCT01773408	
		RO5503781 with abiraierone	Phase I/II in advanced prostate cancer (recruiting)	n/a	CRUKE/12/032	
	Spiro-oxindole	SAR405838	Phase I in advanced solid cancers (active but not recruiting)	n/a	NCT01636479	Sanofi-Aventis
		SAR405838 with pimasertib	Phase I in advanced solid cancers (recruiting)	n/a	NCT01985191	
	Imidazothiazole	DS-3032b	Phase I in advanced solid cancers (recruiting)	n/a	NCT01877382	Daiichi Sankyo
	Dihydroisoquinolinone	CGM-097	Phase I in advanced solid tumors (recruiting)	wtp53	NCT01760525	
	n/a	HDM201	Phase I in advanced solid and hematological cancers (recruiting)	wtp53	NCT02143635	Novartis
		HDM201 with ribociclib	Phase Ib/II in liposarcoma (recruiting)	wtp53	NCT02343172	
	Piperidines	MK4828 with cytarabine	Phase I in acute myelogenous leukemia (terminated)	n/a	NCT01451437	Merck
	Piperidinone	AMG232	Phase I in advanced solid cancers and multiple myeloma (recruiting)	n/a	NCT01723020	Amgen
		AMG 232 with trametinib and dabrafenib	Phase Ib/IIa in metastatic melanoma (recruiting)	n/a	NCT02110355	
	Pyrrolidine	RG7388	Phase 1 in polycythemia vera and essential ihrombocythemia (recruiting)	n/a	NCT02407080	Pegasys
Stapled peptide MDM2/X inhibitor	Peptide	ALRN-6924	Phase I in advanced solid cancers (recruiting)	wtp53	NCT02264613	Aileron

*Data extracted from http://www.clinicaltrials.gov, accessed 1st December 2015*.

### Cellular Responses to Increased p53

Increased cellular p53 protein levels, resulting from MDM2/X inhibition, lead to a number of effects that can be simplified into the broad categories of cell cycle arrest and apoptosis. The decision between these two pathways is governed by the level and duration of p53 induction. Lower and cyclical levels of p53 induce arrest, while sustained levels of elevated p53 expression promotes death (35). Cell cycle arrest is primarily achieved through transcriptional activation of p53 target genes, primarily p21 and GADD45, which block the activity of cyclin-dependent kinases (Cdk) and cause arrest in G1/S (36) and G2 phases, respectively (37). Interestingly, upregulation of p53 during mitosis does not delay mitotic progression, but it is an important requirement for arresting and eliminating aberrant polyploid cells in the subsequent G1 phase (38, 39). Continued p53 expression occurs when the damage or stress incurred cannot be repaired or resolved. These stresses continue to generate a signaling cascade (e.g., ATM/ATR, Chk1/2) that leads to the continued stabilization of p53, and subsequently allows the accumulation of pro-apoptotic p53 targets, including PUMA, Noxa, and Bim within the cell (40, 41). Once these proteins accumulate to sufficient levels, they trigger apoptosis (42, 43).

## MDM2/X Inhibitors in Clinical Trials

The majority of MDM2-targeted therapies currently in clinical development are small molecule inhibitors (Table 1). These have been crystallographically resolved and comprise derivatives that bind to MDM2 by mimicking Phe19, Trp23, and Leu26, which are key residues engaged by p53. ALRN-6924 (Aileron Therapeutics) belongs to a different class of therapeutics, which are stapled peptides designed to disrupt p53 interaction with both MDM2 and MDMX. A number of these compounds are also being evaluated clinically in combination with cytotoxics (doxorubicin and cytarabine), and also molecular-targeted therapies, including ribociclib (CDK4/6 inhibitor), dabrafenib (BRAF inhibitor), trametinib, and pimasertinib (MEK1/2 inhibitors). A number of these trials have excluded patients with p53 mutant tumors; however, the majority have not defined a clear biomarker for selection criteria, in keeping with the primary end points of safety and tolerability. It is of interest that a number of these phase 1 trials have yet to be reported even though accrual was started over 3 years ago, which is unusually long in a phase 1 setting.

RG7112 is the most developed in this class of compounds, and preclinical studies demonstrate strong binding to MDM2, and effective apoptosis, particularly in MDM2-amplified tumors (44). One of first clinical trials reported was in patients with liposarcoma, a tumor characterized by a high proportion of MDM2 gene amplification and wild-type p53 (45). The primary end point in this small neoadjuvant study of 20 patients was to assess tumor biomarkers of p53 pathway activation and cell proliferation. The results demonstrated an increase in intratumoral p53, p21, and macrophage-inhibitory cytokine 1 (MIC1, a secreted protein product of p53) concentrations, an increase in MDM2 mRNA expression and a small decrease in Ki-67 positive cells in the treated compared to the pretreated samples. Clinically, the results were modest, with one partial response and stable disease in 70% of the cohort. Importantly, there were serious adverse events (grade 3 or 4) experienced by 40% of the patients, the majority of which were hematological in nature.

RG7112 has also been evaluated in a phase 1 trial of patients with relapsed/refractory leukemia, such as AML, ALL, CML, and CLL (46). The most common toxicities were gastrointestinal and hematological in nature, 22% of patients experiencing grade 3 and 4 febrile neutropenia. There was clinical activity, particularly in the AML cohort, whereby 5 out of 30 evaluable patients achieved either a complete or partial response, and another 9 patients had stable disease. These numbers suggest useful single agent clinical activity, given the refractory nature of their disease to other therapies. MDM2 inhibition resulted in p53 stabilization and transcriptional activation of p53 target genes. Interestingly, two patients who had p53 mutations (G266E and R181L) also responded to RG7112 in this trial. The G266E is a gain-of-function (GOF) p53 mutation that upregulates CXC-chemokine expression and enhances cell migration (47), while R181L is capable of inducing MDM2 and instigating a cell cycle arrest, but not apoptosis (48). Consequently, these mutants (G266E and R181L) may still be sensitive to MDM2/X inhibitors, and hence patients with these mutations may benefit from these inhibitors.

Assessing the effects that MDM2/X inhibitors in the context of the various GOF p53 mutants will be of significant importance, as MDM2/X inhibition has the potential to increase the levels of GOF p53 mutants. Several GOF mutants have been shown to increase cell proliferation, metabolism, invasion, and chemoresistance in cancer cells (49–53). Consequently, inhibition of MDM2/X could place selective pressure on cancer cells with GOF p53 mutations, driving the clonal evolution of more aggressive cancer cells and exacerbating tumor growth and metastasis in patients. Alternatively, a recent preclinical study demonstrated that the novel small molecule NSC59984 activates p73, resulting in an MDM2-dependent degradation of GOF p53 and subsequent inhibition of tumor growth (54). Other possible explanations for the varied patient response include multiple clones being present with the tumor (only some of which are mutant), a retention of one wild-type allele, certain p53 mutations may still have functional p53 activity (55). Taken together, it is clear that much more work needs to be done to clearly identify biomarkers to improve patient selection for clinical trials of MDM2/X inhibitors. Furthermore, understanding the heterogeneity of p53 expression and the specific mutations within a patient’s tumor prior, during and post treatment will also be of considerable importance for determining the suitability of treatment with MDM2/X inhibitors.

The clinical effect of MDM2 inhibitors on p53 reactivation, range from cytostasis to apoptosis, and a combination strategy may be more efficacious in certain contexts. Preclincal modeling with nutlin-3a has demonstrated improved anti-cancer activity in combination with cytotoxic- and molecular-targeted therapies, in different tumor types (45); however, the toxicity profile of the combination partner is a critical determinant of the success of such an approach clinically. The high incidence of hematological toxicities in the clinical trials of RG7112 would suggest that therapies with an overlapping side effect profile would not be suitable as combination partners (45, 46). A number of clinical trials combining MDM2 inhibitors with cytotoxics have completed accrual but have yet to be reported (Table 1).

## Toxicities

A concern of p53 reactivating therapies is its effect on normal cells. These include the stabilization of p53 resulting in increased apoptosis in these cells. This was reflected in the clinical trial of RG7112 in lipoma, whereby the most common toxicity was hematological in nature, with a reported 30% of patients experiencing grade 4 neutropenia, and 15% experiencing sometimes prolonged grade 4 thrombocytopenia (45, 46). Whether hematologic toxicity correlates with prior exposure to genotoxic therapies is not known. There are also reports of an increased incidence of p53 mutations following prolonged nutlin-3a exposure (56), and concerns about this effect on the development of new cancers (57). Other potential off-target effects on MDM2 inhibitors include the loss of its ability to ubiquitinate other proteins, such as the steroid hormone receptors [estrogen receptor (ER) and androgen receptor (AR)] and Rb, as well as interference with MDM2’s role in DNA repair and modifying chromatin structure (58). The clinical relevance of these potential long-term toxicities have not been reported in the current early phase trials.

## Conclusion/Perspective

Protein–protein interactions, once considered to be a major hurdle to p53 therapeutic development, can now be targeted with a growing number of small molecule inhibitors and stapled peptides. The strategies to overcome this Achilles heel in many cancers are increasingly varied, and build upon an understanding of the crystallographic structure of p53 and its interactions with its major inhibitors. Most of the major pharmaceutical companies have one or more lead compounds targeting MDM2/X, and many of these have only recently progressed from preclinical development into early phase clinical trials.

The effect of MDM2/X-targeting therapies range from cytostasis to apoptosis, and combinatory approaches with other cytotoxic therapies or therapies that target other major oncogenic pathways are logical approaches, and may allow for lower and better tolerated doses of both drugs to be administered. For example, in p53 mutant tumors, protection of normal cells can be achieved by triggering p53-dependent cytostatic effects with short, pulsed exposure to MDM2 inhibitors. This cyclotherapy can reduce the toxic side effect of chemotherapy in these p53 mutant patients (59). Alternatively, recent preclinical evidence has demonstrated that inhibition of MDM2 with nutlin-3a prevents repair of DNA damage, providing synthetic lethality with genotoxic agents, such as cisplatin (60). Importantly, this effect was independent of p53 status and could provide a rational for examining MDM2 combination therapy in p53 mutant patients. Getting the therapeutic index right is critical in patients. It is not surprising that hematological toxicities have been the most commonly reported and dose-limiting toxicities in the trials reported so far (45, 46). Long-term follow-up is also critical to evaluate for the clinical relevance of the potential effects of an increase in p53 mutations and other off-target effects of this class of compounds.

The three major biomarkers that have been used to evaluate therapeutic responses to MDM2/X inhibitors are p53 status, MDM2, and MDMX levels. Interestingly, the over expression of MDM2, MDMX, or mutation of p53 are often mutually exclusive. For example, liposarcoma, which is one of the first tumors in which MDM2 inhibitors have been evaluated (45), shows highly significant tendency toward mutual exclusivity (*p*-value <0.001) between overexpression of MDM2 (19%) and p53 mutation (12%) (Figure 1A) (5). Other tumors with similar trends of exclusivity include glioblastoma multiforme, melanoma, bladder, lung andenocarcioma, prostate, and ER-positive breast cancers. These tumors present an obvious starting point for trialing MDM2/X inhibitors in patients. The high rate of MDM2 overexpression in prostate and ER-positive breast cancers, and the ability of MDM2 inhibitors to ubiquitinate steroid hormone receptors, has led to the evaluation of this class of drugs in combination with endocrine therapies (CRUKE/12/032). It has also been shown that estradiol modulates a subset of p53 and ER target genes that can predict the relapse-free survival of patients with ER-positive breast cancer, and that p53 activation with nutlin in combination with fulvestrant, a selective ER degrader, led to a greater degree of apoptosis *in vitro* (61).

Given the risk of mutations in p53 driving resistance to MDM2/X inhibitors, additional biomarkers need to be identified to maximize the chances of clinical success. This is highlighted by evidence that p53 mutation status as currently measured clinically, may not be an accurate representation of functional p53 activity (46). In support, the recent discovery that MDM2 inhibitor sensitivity could be predicted by a panel of 13 p53 transcriptional target genes (62) was subsequently shown to be based on a significant number of miss-classified p53 mutant cell lines (63). Removal of these lines unfortunately abolished the predicative power of the gene signature. An alternative approach would be to select for tumors with MDM2 amplification given the mutual exclusivity of p53 mutations and MDM2 amplification (64). However, MDM2 and MDMX have different and cooperative inhibitory effects on p53 activity, and therefore inhibitors of one may not be as effective in the setting of raised levels of the other protein (23). Thus, these biomarkers, while logical in their choice, unless further improved upon, may potentially exclude patients who may benefit from these therapies.

## Author Contributions

All authors contributed to the preparation and writing of the manuscript.

## Conflict of Interest Statement

The authors declare that the research was conducted in the absence of any commercial or financial relationships that could be construed as a potential conflict of interest.
